# Environmental Factors Contribute to β Cell Endoplasmic Reticulum Stress and Neo-Antigen Formation in Type 1 Diabetes

**DOI:** 10.3389/fendo.2017.00262

**Published:** 2017-09-29

**Authors:** Meghan L. Marré, Jon D. Piganelli

**Affiliations:** ^1^Division of Pediatric Surgery, Department of Surgery, Children’s Hospital of Pittsburgh, University of Pittsburgh, Pittsburgh, PA, United States

**Keywords:** type 1 diabetes, β cell, environmental factors, endoplasmic reticulum stress, posttranslation modification, neo-antigen, autoimmunity

## Abstract

Type 1 diabetes (T1D) is an autoimmune disease in which immune-mediated targeting and destruction of insulin-producing pancreatic islet β cells leads to chronic hyperglycemia. There are many β cell proteins that are targeted by autoreactive T cells in their native state. However, recent studies have demonstrated that many β cell proteins are recognized as neo-antigens following posttranslational modification (PTM). Although modified neo-antigens are well-established targets of pathology in other autoimmune diseases, the effects of neo-antigens in T1D progression and the mechanisms by which they are generated are not well understood. We have demonstrated that PTM occurs during endoplasmic reticulum (ER) stress, a process to which β cells are uniquely susceptible due to the high rate of insulin production in response to dynamic glucose sensing. In the context of genetic susceptibility to autoimmunity, presentation of these modified neo-antigens may activate autoreactive T cells and cause pathology. However, inherent β cell ER stress and protein PTM do not cause T1D in every genetically susceptible individual, suggesting the contribution of additional factors. Indeed, many environmental factors, such as viral infection, chemicals, or inflammatory cytokines, are associated with T1D onset, but the mechanisms by which these factors lead to disease onset remain unknown. Since these environmental factors also cause ER stress, exposure to these factors may enhance production of neo-antigens, therefore boosting β cell recognition by autoreactive T cells and exacerbating T1D pathogenesis. Therefore, the combined effects of physiological ER stress and the stress that is induced by environmental factors may lead to breaks in peripheral tolerance, contribute to antigen spread, and hasten disease onset. This Hypothesis and Theory article summarizes what is currently known about ER stress and protein PTM in autoimmune diseases including T1D and proposes a role for environmental factors in breaking immune tolerance to β cell antigens through neo-antigen formation.

## Introduction

Type 1 diabetes (T1D) is a chronic autoimmune disease in which insulin-producing pancreatic islet β cells are targeted and destroyed by autoreactive immune cells. Autoimmune recognition of β cell antigens leads to decreased β cell mass and to the subsequent decline of insulin-mediated regulation of glucose levels in the blood. Eventually, too few β cells remain to meet the demand for insulin to maintain normal blood glucose levels. This insufficient insulin secretion leads to chronic hyperglycemia and T1D.

Type 1 diabetes is strongly associated with a genetic predisposition to autoimmunity that is conferred by single-nucleotide polymorphisms (SNPs) and gene variants found at many genetic loci. In particular, SNPs and variants in genes associated with both the innate and adaptive branches of the immune system cause failures of central and peripheral tolerance that eventually lead to autoimmune targeting of β cells. Of these loci, polymorphisms in the major histocompatibility complex (MHC) locus are most strongly associated with T1D onset (1–3). MHC proteins are crucial to central tolerance, because the antigens they present during T cell development in the thymus determine which T cells survive selection. This process directly shapes the mature adaptive immune repertoire. Strongly autoreactive T cells should be deleted upon encountering self-antigen presented by MHC during selection (4), but in individuals expressing MHC polymorphisms associated with autoimmunity, autoreactive T cells successfully mature and exit the thymus (5, 6). If peripheral tolerance mechanisms also fail, these autoreactive T cells become activated when they encounter β cell antigens in pancreatic lymph nodes. This autoimmune response destroys pancreatic β cells and ultimately causes T1D.

To better understand the processes by which the autoimmune response leads to T1D, and to identify the β cell proteins that are targeted by autoreactive T cells, researchers have studied the non-obese diabetic (NOD) mouse. These mice develop a spontaneous autoimmune diabetes that is similar in many ways to the human disease. These similarities include genetic susceptibility at the MHC locus and other immune-related loci, intra-islet infiltration of autoreactive immune cells as disease progresses, and ultimate β cell destruction (7–9). The β cell autoantigens identified using this murine model include preproinsulin (10), glutamic acid decarboxylase 65 (GAD65) (11), islet-specific glucose-6-phosphatase catalytic subunit-related protein (IGRP) (12), chromogranin A (CHgA) (13), islet amyloid polypeptide (14), zinc transporter 8 (15), and 78 kDa glucose-regulated protein (GRP78) (16) (Table 1). Subsequent studies confirmed the relevance of these autoantigens to human T1D (17–23) (Table 1). In addition, several additional autoantigens have been identified in humans but not yet confirmed in NOD mice, including tyrosine phosphatase-like insulinoma antigen 2 and IA-2β (also known as phosphatase homolog of granules from rat insulinomas) (24, 25), and islet cell autoantigen 69 (26) (Table 1).

**Table 1:** β Cell autoantigens identified in murine and human T1D.

Autoantigen	Species	Reference
Preproinsulin	Mouse	(10)
	Human	(20)

Glutamic acid decarboxylase 65	Mouse	(11)
	Human	(17)

IGRP	Mouse	(12)
	Human	(22)

Chromogranin A	Mouse	(13)
	Human	(19)

Islet amyloid polypeptide	Mouse	(14)
	Human	(18)

Zinc transporter 8	Mouse	(15)
	Human	(21)

78 kDa glucose-regulated protein	Mouse	(16)
	Human	(23)

IA-2, IA-2β	Human	(24, 25)

Islet cell autoantigen 69	Human	(26)

The immunogenicity of these β cell autoantigens has long been attributed to failures in the mechanisms that govern immune tolerance to self-peptides. While this likely remains true, seminal studies conducted by several laboratories demonstrated that many of these β cell peptides undergo posttranslational modification (PTM). These studies propose that aberrant PTM of these β cell proteins generates so called “neo-antigens” that are then recognized as non-self by immune cells (16, 19, 23, 27–32), hastening the break in tolerance and exacerbating immune targeting and destruction of β cells. However, most of these studies did not explore the cellular processes that lead to PTM of these proteins in the context of β cell function and biology.

**Figure 1:**
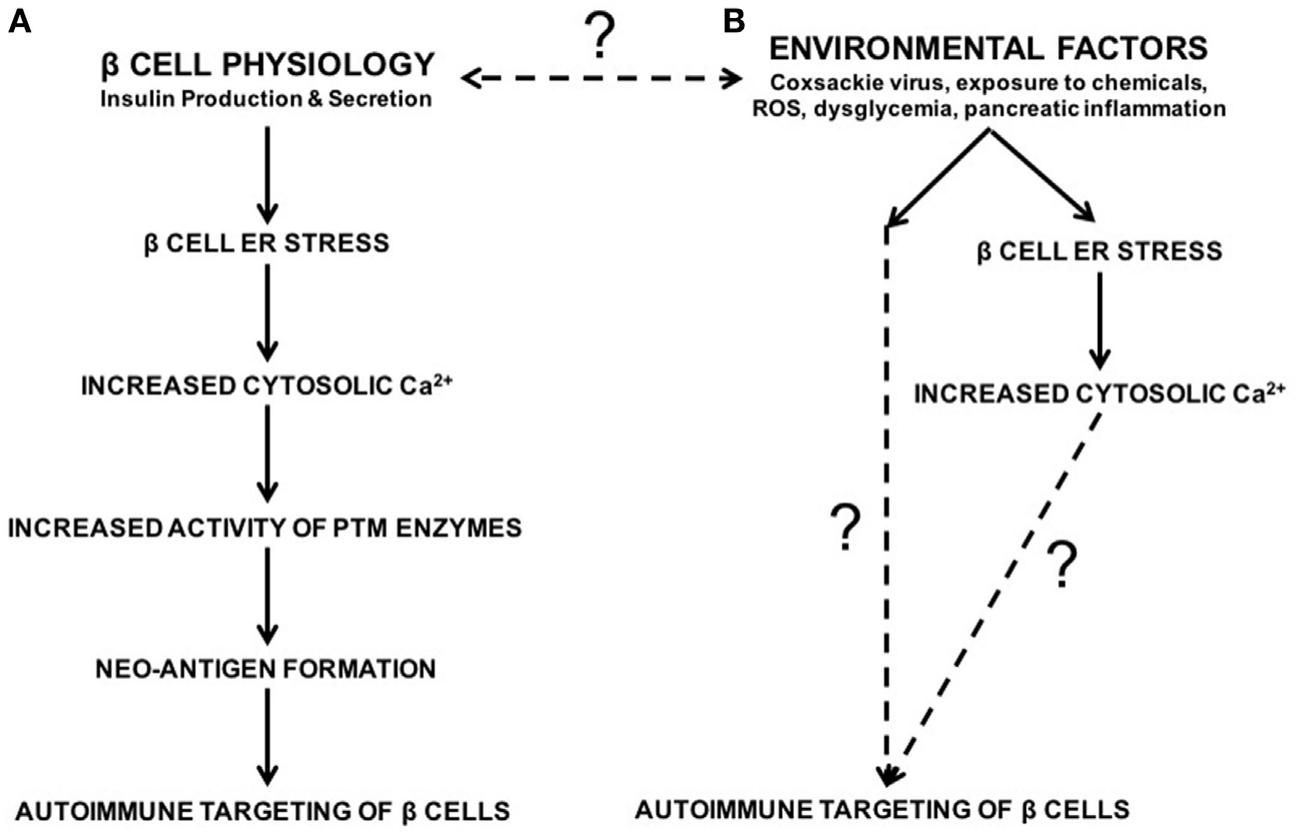
The roles of β cell physiology and environmental favors in the autoimmune targeting of β cells in type 1 diabetes (T1D). **(A)** Normal β cell secretory physiology causes inherent endoplasmic reticulum (ER) stress, which in turn results in a release of Ca^2+^ from the ER into the cytosol. We have previously demonstrated that ER stress and its Ca^2+^ efflux lead to increased activity of Ca^2+^-dependent posttranslational modification enzymes, formation of neo-antigens, and β cell immunogenicity (32). **(B)** In addition, many environmental factors are associated with T1D onset, such as viral infection, exposure to chemicals and reactive oxygen species, dysglycemia, and pancreatic inflammation. Although the mechanisms by which these factors lead to autoimmune targeting of β cells remain unknown, these environmental factors all cause β cell ER stress and Ca^2+^ efflux. Whether the ER stress and Ca^2+^ efflux caused by these environmental factors contributes to T1D onset, and whether this ER stress cooperates with physiological ER stress to generate neo-antigens, remain unknown.

To address this question, our laboratory demonstrated that endoplasmic reticulum (ER) stress in the β cell leads to the activation of PTM enzymes and the modification of β cell proteins, which in turn leads to increased recognition of these β cells by diabetogenic T cells (32). ER stress in the β cell originates from various sources. For instance, the normal function of β cells (to produce and secrete insulin) causes ER stress (32–42). We demonstrated that this inherent physiological ER stress is sufficient to activate PTM enzymes and to generate β cell immunogenicity (32) (Figure 1). In addition, many of the environmental factors associated with T1D onset such as viral infection (43–48), chemicals (32, 49–51), reactive oxygen species (ROS) (52–55), dysglycemia (56), and inflammation (57–59) may cause β cell ER stress (Figure 1). Therefore, any of these environmental factors has the potential to enhance autoimmune targeting of β cells through the generation of ER stress- and PTM-dependent neo-antigens (32, 60, 61). However, the mechanisms by which these factors hasten T1D onset, and whether the ER stress they cause cooperates with that caused by β cell physiology, remain unknown (Figure 1).

Here, we review what is known about β cell ER stress, neo-antigen formation, and the progression to pathology in T1D. We also review the role that the environmental factors associated with T1D may play in exacerbating β cell ER stress. Finally, we discuss the evidence supporting our novel hypothesis that environmental factors converge with β cell physiology to increase ER stress above a putative threshold. According to our “threshold hypothesis,” ER stress must be sufficiently severe or prolonged to allow for the generation of PTM-dependent neo-antigens. We hypothesize that the convergence between β cell physiology and exposure to environmental factors increases ER stress above this threshold, leading to neo-antigen formation, β cell immunogenicity, and ultimately to the onset of T1D.

## ER Stress and the Unfolded Protein Response (UPR)

The ER is primarily responsible for the proper folding and modification of proteins that are membrane bound or destined for secretion. Therefore, the ER lumen contains the molecular chaperones and the environment necessary for protein folding and PTM, including sufficient levels of adenosine triphosphate, an oxidizing environment to support disulfide bond formation, and millimolar concentrations of calcium (Ca^2+^) (62). Proteins that are folded and modified properly exit the ER and are shuttled to their intended intra- or extracellular locations. However, proteins that become misfolded cannot exit the ER lumen. The accumulation of misfolded or aberrantly modified proteins causes ER stress.

Endoplasmic reticulum stress induces the UPR, which functions in two main modes: the adaptive UPR and the terminal UPR (63, 64). The adaptive UPR occurs early in ER stress and functions largely to alleviate ER stress and restore normal cellular homeostasis through three signaling cascades, each of which begins with the activation of protein sensors of stress in the ER membrane (65). First, protein kinase RNA (PKR)-like ER kinase (PERK) activates a signaling cascade that inhibits mRNA translation and reduces the protein burden in the lumen of the ER (66, 67). Second, activating transcription factor 6 signaling leads to increased production of new molecular chaperones to aid with the folding of accumulated misfolded proteins (68). And third, the signaling pathway initiated by inositol-requiring protein 1 increases expression of chaperones for protein folding and of proteins involved in lipid synthesis to increase ER volume (69, 70). Together, these branches of the UPR work to facilitate the proper folding of proteins that have accumulated, and also reduce the entrance of additional non-chaperone proteins into the ER lumen. In these ways, the adaptive UPR acts to allow the ER to return to normal homeostasis.

Although the adaptive UPR aims to protect the cell from the negative effects of ER stress, ER dysfunction that is excessive or extended may overcome these cytoprotective mechanisms. Under these conditions, the terminal UPR activates proapoptotic processes (71–73) leading to death of the affected cell. However, long before apoptosis pathways are activated, even temporary ER stress and the adaptive UPR may have important consequences for cellular function and physiology.

All cells undergo periods of increased protein production, which increases the ER burden, leading to misfolding or aberrant modification of nascent proteins, and ultimately to ER stress and UPR activation. However, secretory cells, due to their normal physiology, are uniquely susceptible to ER stress. These cells must produce not only the proteins necessary for normal cellular maintenance, but also the proteins to be secreted and the proteins that comprise the secretory pathway itself. Even with a larger ER volume and greater numbers of chaperones to account for this increased demand (74), the secretory function of these cells leads to significantly increased ER burden and stress.

**Figure 2:**
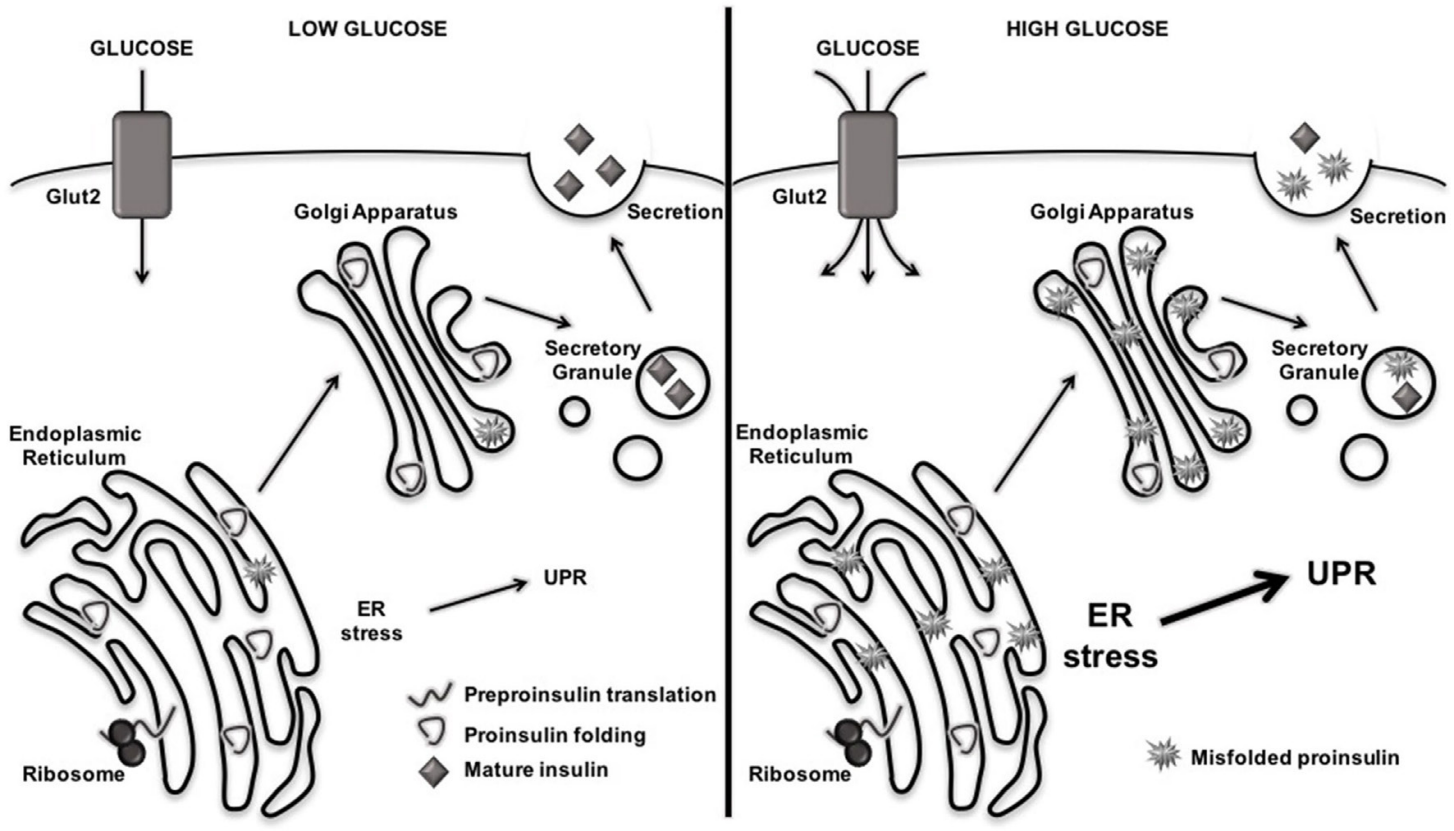
Rising blood glucose increases misfolding of proinsulin and endoplasmic reticulum (ER) stress levels. When blood glucose levels are low, preproinsulin is translated, properly folded and modified in the ER, and secreted as mature insulin into the extracellular space. When blood glucose levels rise, β cells increase production of preproinsulin, flooding the ER lumen with one million molecules per minute that require folding and disulfide bond formation. This increased protein burden in the ER leads to misfolding of proteins and aberrant posttranslational modification, which further exacerbates ER dysfunction and activates the unfolded protein response (UPR).

Like other secretory cells, β cells undergo naturally high levels of ER stress due to their normal physiological role of insulin production and secretion (32–42). Indeed, increased ER stress, and its consequences for protein folding, occurs as a direct consequence of glucose sensing (37, 38). In response to increased glucose concentrations, β cells upregulate the translation of preproinsulin by 50-fold, reaching a production rate of one million molecules of preproinsulin per minute (75). These one million molecules of preproinsulin inundate the ER lumen for folding and the formation of three disulfide bonds per molecule, causing tremendous ER stress. Under these conditions, many of the insulin molecules produced by β cells become misfolded or incorrectly modified (75) (Figure 2). In addition to this inherent ER stress due to normal physiology, β cell ER stress may rise due to exposure to the environmental factors that are associated with T1D onset (32, 43–61) (Figure 1). Under these circumstances, β cell ER stress may rise above physiological levels.

Heightened β cell ER stress does not necessarily activate the terminal UPR or suggest β cell exhaustion or impending death as observed in some models (63, 76–80). Rather, β cells exhibit naturally high ER stress very early and activate the adaptive UPR long before β cell death. In a study using a reporter mouse in which green fluorescent protein is expressed with the activation of the UPR, the pancreas exhibited the highest ER stress of all tissues examined, and did so as early as day 16 of life (81). In spite of the observed ER stress and UPR activation, these mice (on the C57Bl/6 background) never succumbed to loss of β cell mass and diabetes (81). These data confirm that high levels of β cell ER stress does not necessarily activate the terminal UPR and lead to β cell failure and death. Indeed, the β cells in most individuals resolve ER stress through the proper function of the adaptive UPR and therefore maintain healthy and functional β cell mass throughout their lifetimes. Therefore, β cell death is not the only consequence of ER stress. We hypothesize that lower and more transient ER stress, and the activation of the adaptive UPR, may have consequences for β cell function and for the autoimmune targeting of β cells much earlier.

## ER Stress Affects Ca^2+^-Dependent Cellular Functions

In addition to its role in the folding and modification of new proteins, the ER contains the largest store of intracellular Ca^2+^ and is an important organelle for regulating Ca^2+^ concentrations, and therefore Ca^2+^-dependent processes, throughout the cell (82). One consequence of ER stress is the release of Ca^2+^ from the ER lumen into the cytosol. This Ca^2+^ efflux has important consequences for cellular physiology.

In the ER, millimolar concentrations of Ca^2+^ are necessary for proper protein folding and modification (62). Indeed, molecular chaperones that assist in protein folding and protein disulfide isomerases that facilitate the formation of disulfide bonds depend on these high Ca^2+^ concentrations (83, 84). These high concentrations of Ca^2+^ are maintained by sarco/endoplasmic reticulum Ca^2+^ ATPases (SERCA) pumps in the ER membrane that actively transport Ca^2+^ from the cytosol into the ER. When Ca^2+^ leaves the ER lumen during ER stress, the function of these proteins also decreases, further inhibiting protein folding and modification and contributing to greater ER dysfunction (85).

In the cytosol, Ca^2+^ is required for the regulation of normal cellular processes such as metabolism, vesicular trafficking, protein secretion, mRNA transcription, and apoptosis (86). To achieve the necessary cytosolic concentrations, Ca^2+^ is released from the ER by the ryanodine receptor and inositol 1,4,5-trisphosphate receptor channels. Under conditions of ER stress, the efflux of Ca^2+^ from the ER lumen increases cytosolic concentrations above normal physiological levels. This increased cytosolic Ca^2+^ can be deleterious for cellular function. For example, increased cytosolic Ca^2+^ can initiate apoptosis through activation of caspase-dependent cell death pathways (87, 88) or mitochondria-dependent pathways (89–92).

It is clear, then, that ER stress greatly affects Ca^2+^-dependent cellular functions. While the adaptive UPR works to relieve ER stress, cytosolic Ca^2+^ still increases before ER homeostasis is regained. β cells, which are particularly susceptible to ER stress, are therefore also prone to the dysregulation of cellular processes following even a temporary efflux of Ca^2+^ from the ER. In addition, the environmental factors associated with T1D onset also lead to increased cytosolic Ca^2+^ (32, 43–61) (Figure 1). Therefore, we propose that the combination of physiological ER stress and that derived from environmental factors, even if transient, may have consequences for β cell health and function.

## ER Stress Activates Cytosolic PTM Enzymes

Transient ER stress and increased cytosolic Ca^2+^ concentrations can activate cytosolic Ca^2+^-dependent enzymes, including those that mediate PTM. Activation of these PTM enzymes can have significant implications for proteins being folded in the ER. In particular, two such PTM enzymes reside in the cytosol and are activated during the ER stress Ca^2+^ flux: tissue transglutaminase 2 (Tgase2) and peptidylarginine deiminase 2 (PAD2).

Tissue transglutaminase 2 is ubiquitously expressed and resides in the cytosol (93). When activated, Tgase2 translocates to several intracellular compartments (94), including the ER (95–97) and secretory granules (98) to modify proteins through two mechanisms (99): first, Tgase2 crosslinks proteins through the formation of ε(γ-glutamyl) isopeptide bonds between glutamine and lysine residues, and second, Tgase2 mediates the deamidation of glutamine residues. Tgase2 plays important roles in the regulation of apoptosis (100, 101), gene expression (93, 102, 103), and cellular adhesion and wound healing (104–107). Of relevance to T1D, Tgase2 is expressed in and functions in β cells (32, 60).

Of the five mammalian PAD isoforms, PAD2 is the most widely expressed and is the isoform expressed in the pancreas (108). PAD2 also resides in the cytosol (109), and, similar to Tgase2, activated PAD2 is recruited to various subcellular compartments for the modification of proteins (110). PAD2 mediates the conversion of arginine to citrulline. This amino acid conversion alters the overall charge and hydrophobicity of the protein (111), causing changes in protein folding and conformation (112). PAD2 plays roles in many cellular functions, including the negative regulation of nuclear factor kappa-light-chain-enhancer of activated B cells activation (113), cytoskeleton disassembly (114), and in the formation of neutrophil extracellular traps (115).

While Ca^2+^-dependent activation of these enzymes is necessary for normal cellular function, these enzymes also contribute to pathology in many diseases.

## PTM Generates Neo-Antigens in Autoimmune Diseases

Protein PTM is necessary for cellular viability and function. However, autoantigens in many different autoimmune diseases such as celiac disease (116), collagen-induced arthritis (117), multiple sclerosis/experimental autoimmune encephalomyelitis (118–121), rheumatoid arthritis (122–127), and systemic lupus erythematosus (128–131) contain PTM, suggesting that these modifications may contribute to breaks in tolerance that exacerbate disease.

Central tolerance is established during T cell development in the thymus. In the thymus, medullary thymic epithelial cells (mTECs) express peptides normally found in peripheral tissues through the function of autoimmune regulator (132–134). When these peptides are presented to developing T cells in the context of MHC molecules, T cells that respond too strongly to these self-peptides are deleted and are thus absent from the mature T cell population (4, 135–137). However, if self-proteins undergo PTM in peripheral tissues, as in the autoimmune diseases listed above, these proteins may be processed and presented differently by peripheral antigen-presenting cells (APCs) than by the mTECs (138). If such modified epitopes were not expressed and presented by mTECs, T cells that recognize these modified epitopes escape negative selection and are present in circulation as mature T cells. When these T cells encounter these neo-antigens in peripheral tissues, they become activated and lead the autoimmune targeting of peripheral tissues.

As with all peripheral tissues, peptides from β cell proteins, including insulin and insulin-like growth factor 2, are presented by mTECs to developing T cells in the thymus (5, 6, 139–142). However, the presence of T cells in the periphery that recognize islet proteins and target β cells suggests the failure of crucial tolerance mechanisms. This failure in central tolerance mechanisms may be explained by the growing body of literature that abnormal PTM increases the immunogenicity of β cell peptides in both murine and human models of T1D (Table 2). These studies have demonstrated that some β cell proteins undergo various modifications including oxidation (28, 143), Tgase2-mediated crosslinking by isopeptide bond (19, 29), Tgase2-mediated deamidation (30–32), PAD2-mediated citrullination (16, 23, 31), the formation of hybrid peptides (144, 145), and the formation of a defective ribosomal insulin gene product (146). Furthermore, the neo-antigens formed by these PTM elicit stronger immune responses than the native proteins (16, 19, 23, 28–31, 143, 145), suggesting an important role for these neo-antigens in precipitating disease onset. These findings have been of great importance to the understanding of T1D pathogenesis, because these studies identified novel autoantigens that are targeted in T1D. However, the mechanisms by which these neo-antigens arise in β cells was not examined.

**Table 2:** Posttranslational modification (PTM)-mediated neo-antigen formation in type 1 diabetes.

Autoantigen	PTM	Reference
Proinsulin	Oxidation	(28, 143)
	Formation of hybrid insulin peptides	(23, 144, 145)
Chromogranin A (WE14)	Crosslinking/isopeptide bond	(19, 29)
Preproinsulin	Deamidation	(30)
Islet cell autoantigen 69	Deamidation	(30)
Zinc transporter 8	Deamidation	(30)
Phosphatase homolog of granules from rat insulinomas	Deamidation	(30)
IA-2	Deamidation	(30)
IGRP	Deamidation	(30)
Glutamic acid decarboxylase 65	Citrullination	(31)
	Deamidation	(30, 31)
78 kDa glucose-regulated protein	Citrullination	(16, 23)
Insulin	Defective ribosomal product	(146)

## β Cell Neo-Antigens Arise During ER Stress

To begin to elucidate how PTM neo-antigens might arise in β cells, our laboratory examined the consequences of β cell ER stress for β cell immunogenicity, since β cells inherently undergo high levels of ER stress (32–42, 60, 81). To do so, we used a model system of β cell recognition by diabetogenic BDC2.5 CD4^+^ T cells. These particular T cells were chosen because they recognize a Tgase2-modified peptide of CHgA (29) and secrete interferon gamma (IFNγ) when they encounter their PTM-dependent antigen.

**Figure 3:**
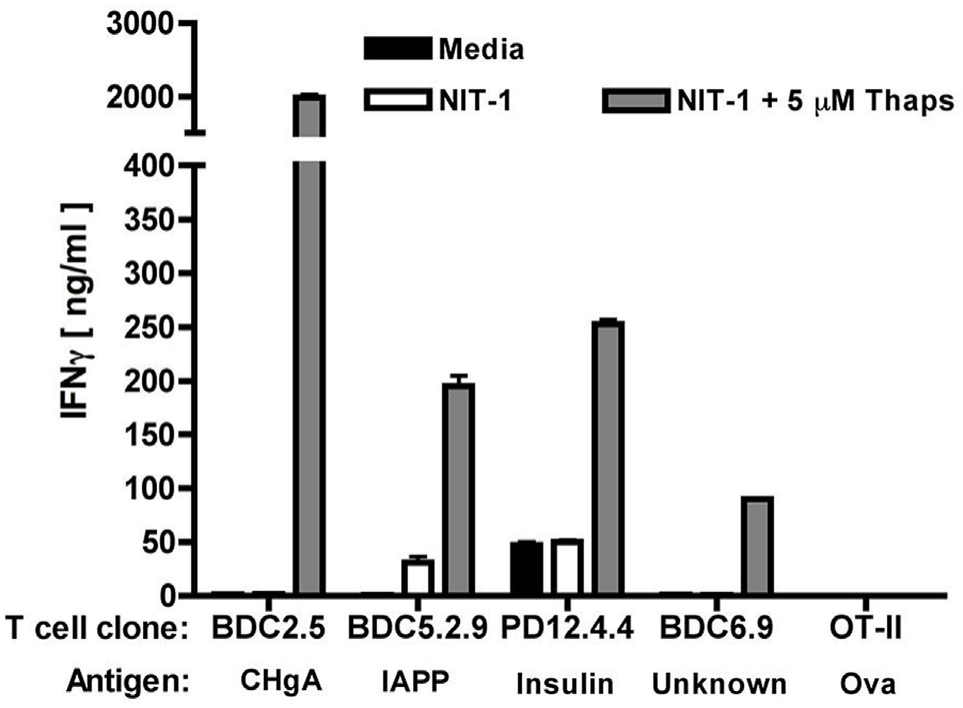
Endoplasmic reticulum (ER) stress increases the immunogenicity of several β cell autoantigens. The immunogenicity of NIT-1 insulinoma cells treated with 5 µM thapsigargin or control for 1 h was assessed by T cell assay. Briefly, T cells (2 × 10^4^), NOD.*scid* splenocytes as antigen-presenting cells (4 × 10^5^), and NIT-1 cells as antigen (1 × 10^3^) were combined in 200 µl in triplicate in 96-well flat-bottom tissue culture plates and incubated at 37°C for 72 h. T_H_1 effector function was determined by measuring interferon gamma (IFNγ) secretion by enzyme-linked immunosorbent assay. Data are mean IFNγ secretion ± SD and are from one representative experiment of three independent experiments. For all specificities examined, NIT-1 cells undergoing ER stress elicited higher effector responses from the T cells, suggesting that ER stress contributes to the modification and greater immunogenicity of each of these proteins.

Our studies demonstrated that, in primary murine islets, ER stress induced by thapsigargin [a widely accepted chemical inducer of ER stress (96, 147, 148)] contributed to heightened cytosolic Ca^2+^ concentrations, increased Tgase2 activity, and increased β cell immunogenicity (32). In fact, murine islets undergoing ER stress elicited greater IFNγ secretion from BDC2.5 T cells (32) and by all other β cell antigen-specific T cells examined (Figure 3), suggesting a role for Ca^2+^-dependent PTM in immunogenicity of many other β cell antigens. This increased immunogenicity was dependent upon both Ca^2+^ and Tgase2-mediated PTM, since chelation of cytosolic Ca^2+^ or decreased expression of Tgase2 reduced this consequence of ER stress (32). These data show that β cell ER stress leads to β cell immunogenicity through Ca^2+^-dependent PTM of endogenous proteins.

Since ER stress is inherent to β cell physiology and function (32–42, 60), we hypothesized that ER stress induced by normal physiology [e.g., dynamic glucose sensing and secretory function (33–42, 60)] may be sufficient to cause Ca^2+^- and PTM-dependent β cell immunogenicity. Indeed, a murine insulinoma (NIT-1) that exhibited low ER stress and immunogenicity was exposed to physiological milieu by transplantation into NOD.*scid* mice. After transplant, these cells exhibited insulin secretion, ER stress, Tgase2 activity, and immunogenicity (32). These data confirm that β cell physiology and insulin secretion contributes to the autoimmune targeting of β cells (60).

**Figure 4:**
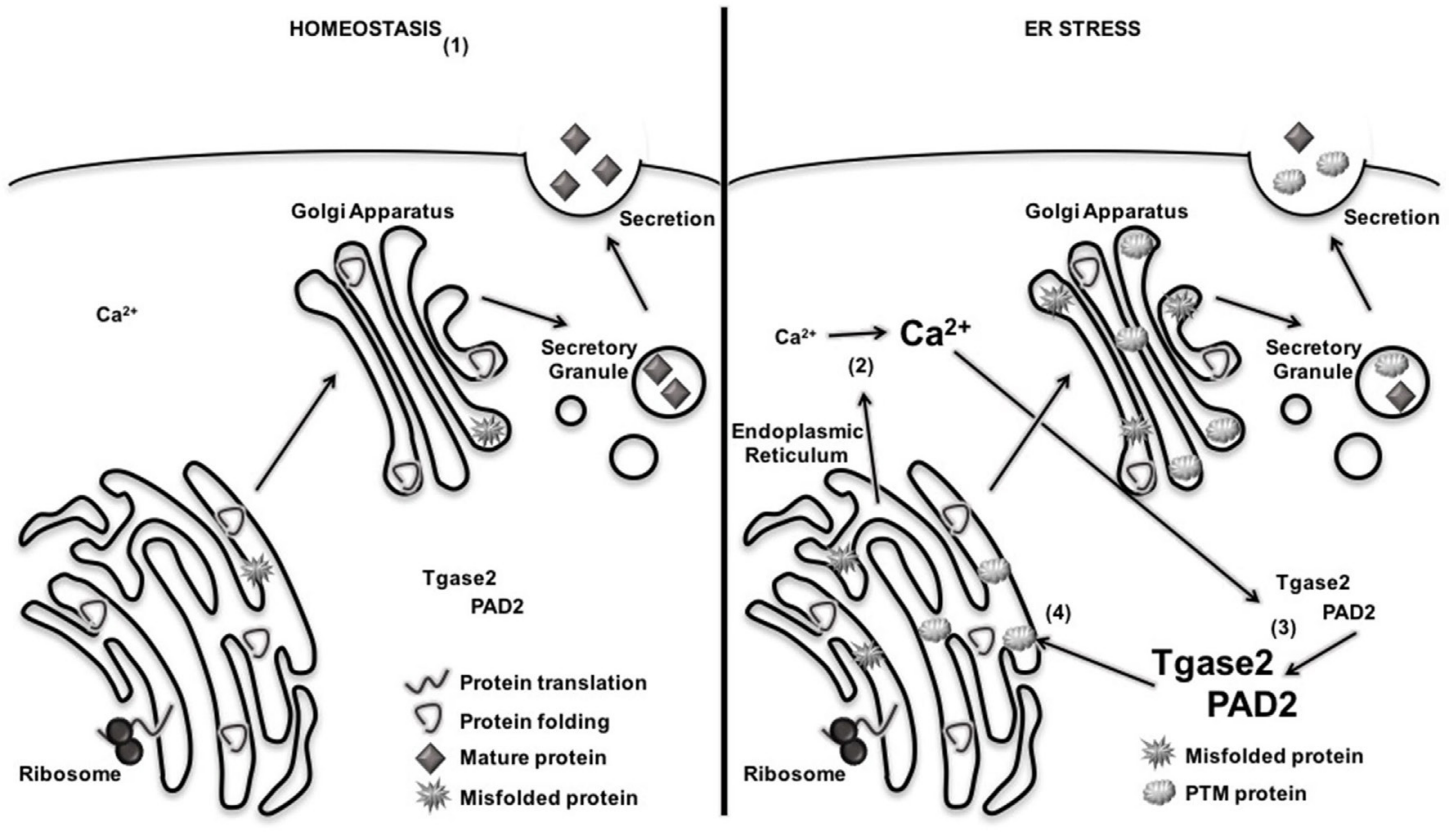
Endoplasmic reticulum (ER) stress increases the activation of Ca^2+^-dependent posttranslational modification (PTM) enzymes and the formation of PTM-dependent β cell neo-antigens. (1) Under homeostatic conditions, proteins are translated, folded, and packaged into secretory granules. Cytosolic Ca^2+^ and PTM enzyme activity remain low. (2) During β cell ER stress, Ca^2+^ stores are released from the ER, increasing cytosolic Ca^2+^. (3) Increased Ca^2+^ concentrations activated Ca^2+^-dependent enzymes tissue transglutaminase 2 (Tgase2) and peptidylarginine deiminase 2 (PAD2). (4) Active PTM enzymes modify nascent proteins. If presented to autoreactive T cells by antigen-presenting cell, modified β cell proteins break tolerance and facilitate immune recognition of β cells.

Many groups have demonstrated an increase in β cell ER stress long before β cell death and T1D onset (79, 81, 149, 150). In fact, relief of ER stress has been proposed as therapeutic opportunity for preventing β cell death and maintaining euglycemia (63, 80, 151, 152). However, most researchers conclude that ER stress leads to β cell death through the terminal UPR and activation of apoptosis pathways (76, 77, 80). Ours was the first study to demonstrate that normal, physiological β cell ER stress and the adaptive UPR contribute to T1D through the formation of β cell neo-antigens. In doing so, we became the first to propose a mechanism by which β cell neo-antigens (Table 2) may occur (Figure 4).

## β Cell Immunogenicity Requires a Threshold of ER Stress

Endoplasmic reticulum stress occurs along a gradient. The burden of unfolded proteins in the ER lumen can vary from mild to severe, resulting in varying degrees of ER dysfunction and stress. This variance in levels of ER stress has important implications for the cellular consequences of ER stress. As discussed earlier, the strength and duration of ER stress-induced UPR signaling is a major factor in determining whether the adaptive UPR or terminal UPR is initiated (63, 64). One explanation may be that the severity and duration of ER stress affects the strength of the Ca^2+^ efflux from the ER lumen and determines whether cytosolic Ca^2+^ concentrations cross a putative threshold. Differences in cytosolic Ca^2+^ concentrations may significantly alter PTM enzyme activity, neo-antigen generation, and β cell immunogenicity.

This “threshold hypothesis” is further supported by literature that demonstrates that Tgase2 and PAD2 remain largely inactive in the cytosol, and activation requires significantly increased concentrations of cytosolic Ca^2+^. In fact, the activation of both enzymes requires Ca^2+^ concentrations up to 100-fold higher than what is necessary for normal cellular physiology and function. Therefore, these enzymes generally become activated only under conditions of cellular distress or dysfunction, such as ER stress (96, 97, 108, 109, 147, 153, 154). Since these PTM enzymes require particular levels of cytosolic Ca^2+^ to become activated, it follows that a particular level of ER stress must be achieved before PTM-dependent neo-antigen formation can occur.

**Figure 5:**
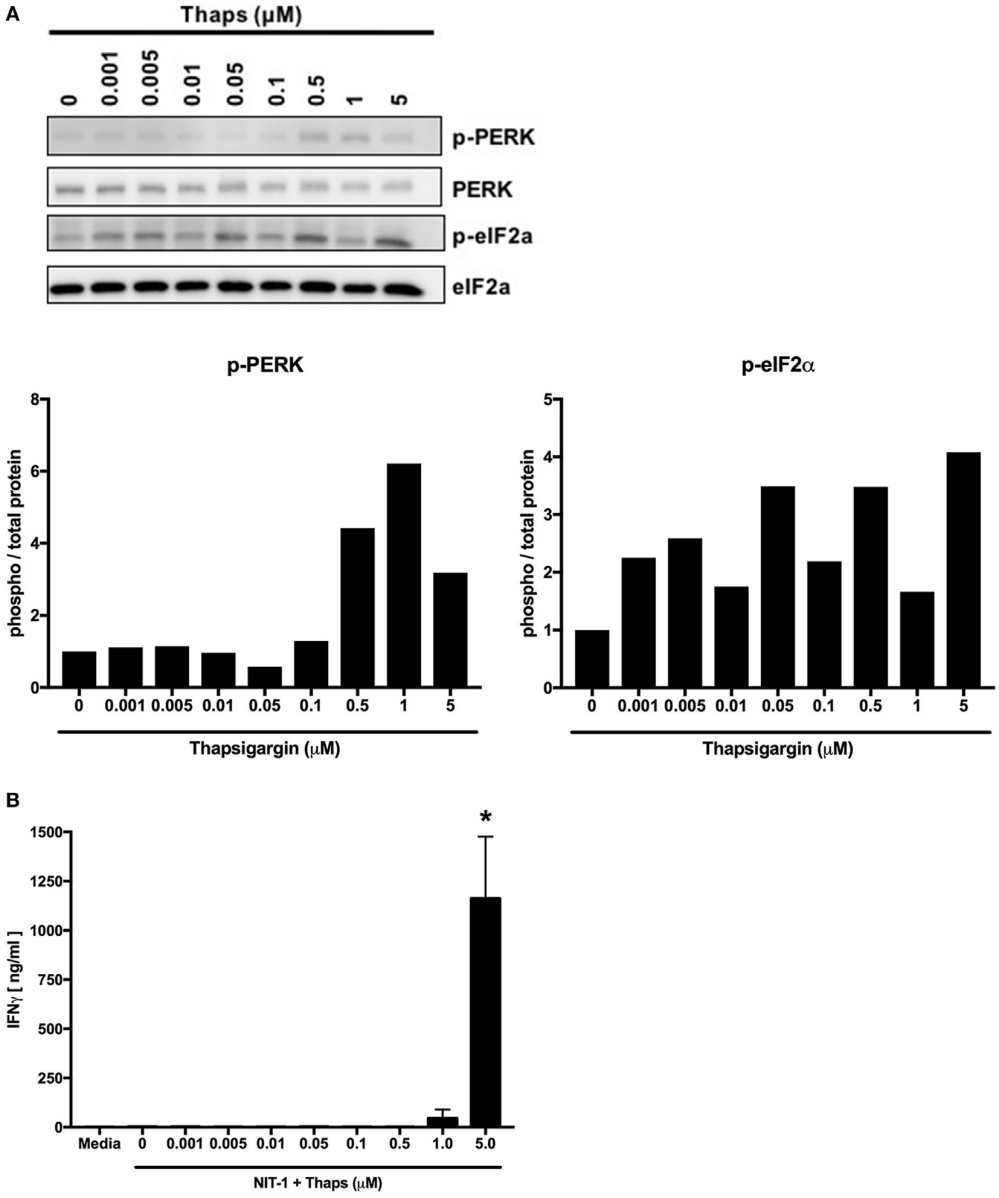
Endoplasmic reticulum stress must increase above a threshold to induce posttranslational modification-dependent immunogenicity. **(A)** NIT-1 insulinoma cells were incubated with increasing concentrations of thapsigargin for 1 h and washed extensively. Cell lysates were analyzed for the phosphorylation of UPR proteins—protein kinase RNA (PKR)-like ER kinase (PERK) and eIF2α. Data are representative of two independent experiments. Densitometry data are phosphorylation levels normalized by total protein and relative to that in control (0 µM) treated cells. **(B)** The immunogenicity of NIT-1 cells treated with increasing concentrations of thapsigargin for 1 h was measured by BDC2.5 T cell assay. Data are mean interferon gamma (IFNγ) secretion ± SEM. *p < 0.05.

Previous work in our laboratory examined whether varying levels of ER stress lead to different degrees of β cell PTM-dependent immunogenicity. NIT-1 cells were incubated with increasing doses of thapsigargin, which increases ER stress and cytosolic Ca^2+^ by inhibiting the SERCA pumps that transport Ca^2+^ from the cytosol into the ER. As expected, thapsigargin induced ER stress and UPR activity in a dose-dependent manner (Figure 5A). The immunogenicity of these cells was examined by the BDC2.5 T cell clone, and T cell effector function was measured by IFNγ as previously described (32). Only the highest dose of thapsigargin elicited detectable IFNγ secretion from the T cells (Figure 5B). Therefore, although lower doses of thapsigargin induced ER stress, the stress (and consequences thereof) at these lower doses was not sufficient to result in β cell immunogenicity.

**Figure 6:**
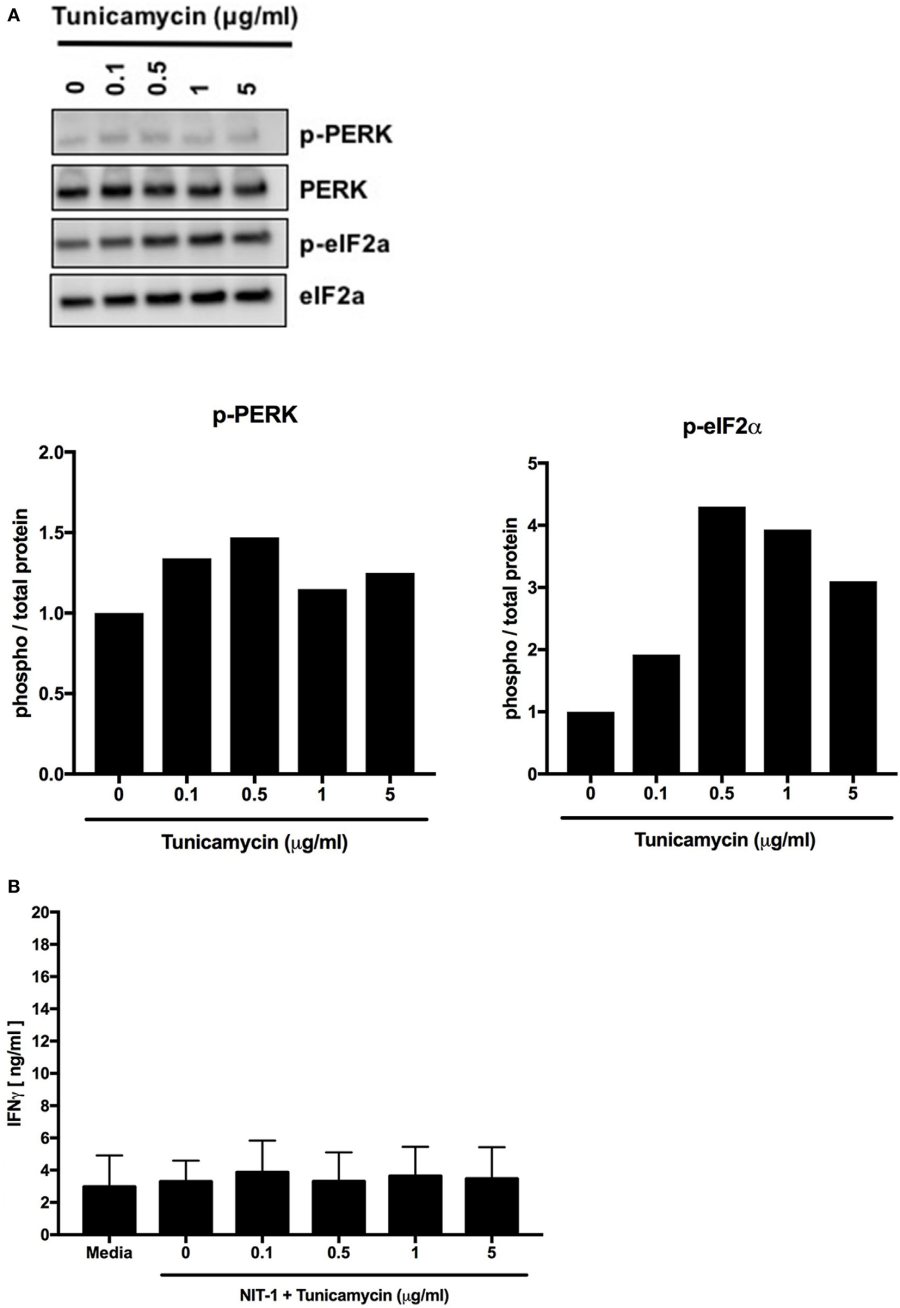
Endoplasmic reticulum stress below a threshold does not induce posttranslational modification-dependent immunogenicity. **(A)** NIT-1 insulinoma cells were incubated with increasing concentrations of tunicamycin for 4 h and washed extensively. Cell lysates were analyzed for the phosphorylation of UPR proteins—protein kinase RNA (PKR)-like ER kinase (PERK) and eIF2α. Data are representative of two independent experiments. Densitometry data are phosphorylation levels normalized by total protein and relative to that in control (0 µg/ml) treated cells. **(B)** The immunogenicity of NIT-1 cells treated with increasing concentrations of tunicamycin for 4 h was measured by BDC2.5 T cell assay. Data are mean interferon gamma (IFNγ) secretion ± SEM.

Tunicamycin is another chemical inducer of ER stress that blocks the initial steps of glycoprotein synthesis in the ER and thus increases the burden of unfolded proteins in the ER lumen (148). Increasing doses of tunicamycin increased ER stress in NIT-1 cells (Figure 6A), but to lesser degrees compared with thapsigargin (Figure 5A). Also, as with lower doses of thapsigargin, the lower ER stress induced by tunicamycin was not sufficient to elicit effector responses from BDC2.5 T cells (Figure 6B). Together, these data serve as further evidence that a particular threshold of ER stress must be reached to achieve PTM-dependent β cell immunogenicity.

## Environmental Factors Associated with T1D Induce Heightened β Cell ER Stress

Every pancreas undergoes ER stress (32, 81), but this stress does not lead to T1D in every individual. In fact, even in those with a genetic predisposition to autoimmunity, T1D may never occur (155) or may occur much later than expected (156, 157). These observations suggest that environmental factors may precipitate disease onset. Indeed, T1D onset is associated with several environmental factors such as viral infection (43–48), chemicals (49–51), ROS (52–55), dysglycemia (56), and inflammation (57–59). Although these environmental factors are thought to exacerbate the autoimmune targeting of β cells and hasten disease onset, the mechanisms by which these environmental factors advance pathology, and whether these factors contribute to PTM-mediated neo-antigen formation, remain unknown (Figure 1).

As discussed earlier, β cell ER stress and Ca^2+^ flux into the cytosol must cross a threshold before Tgase2 and PAD2 can modify β cell proteins to generate neo-antigens and elicit effector responses from diabetogenic T cells. While β cell physiology causes ER stress (32–42) and this ER stress can, under some circumstances generate neo-antigens and immunogenicity (32, 60) (Figure 1), the discrepancy in disease onset in those genetically predisposed to autoimmunity (155–157) suggests that this physiological stress alone may not be sufficient to generate neo-antigens. Interestingly, each of the environmental factors associated with T1D also lead to an increase in β cell ER stress and cytosolic Ca^2+^.

### Coxsackie Virus

Coxsackie virus infection is associated with T1D onset. Recent onset T1D patients have viral RNA in their pancreas and higher titers of antibodies against Coxsackie virus (158, 159). Also, Coxsackie virus infection accelerates disease onset in NOD mice with established insulitis (46, 160–162), suggesting a role for Coxsackie virus in breaking immune tolerance. Studies with BDC2.5 TCR transgenic NOD mice attributed this acceleration to activation of bystander immune cells (46). These data provide a strong link between pancreatic viral infection and broken tolerance. Since BDC2.5 T cells do not recognize a viral protein (29) but rather modified CHgA, activation of BDC2.5 T cells in these mice suggests that Coxsackie virus infection may lead to PTM of endogenous β cell proteins and neo-antigen formation. Indeed, viruses cause neo-antigen generation and exacerbate pathology in other models of autoimmunity (163).

Moreover, Coxsackie virus protein 2B disrupts the ER membrane (164–166), releasing Ca^2+^ from the ER into the cytosol and causing ER stress. We have shown that β cell ER stress contributes to neo-antigen formation and immunogenicity (32). Therefore, it is plausible that Coxsackie virus may raise β cell ER stress and cytosolic Ca^2+^ concentrations above the levels attributed to normal physiology, increasing neo-antigen production through Ca^2+^-dependent PTM.

### Exposure to Chemicals

Exposure of β cells to chemicals such as alloxan and streptozotocin cause the loss of insulin secretion and β cell death (167). For each of these chemicals, β cells experience DNA damage, protein ADP ribosylation (168), and ROS generation (169–171), all of which ultimately lead to apoptosis and significant loss of β cell death. However, before apoptosis pathways are activated, ADP ribosylation and ROS cause misfolding and accumulation of nascent proteins in the ER lumen. As discussed earlier, the accumulation of misfolded and abnormally modified proteins leads to ER stress and release of Ca^2+^ into the cytosol (172, 173).

### Reactive Oxygen Species

Reactive oxygen species, which have the potential to cause irreversible damage to cellular proteins and organelles (174–176), are generated both during normal β cell function (52) and when β cells are exposed to other insults such as Coxsackie virus (177–179). Although antioxidant defenses work to prevent ROS-mediated damage, β cell mitochondria express very low levels of antioxidant enzymes (180–182), making these cells particularly susceptible to ROS-mediated damage. When ROS exceeds the capacity of the cell to scavenge these species, oxidative stress leads to β cell death (183, 184) and ultimately to T1D (52, 54, 180, 185–190). However, before the loss of β cell mass, ROS leads to oxidative modification of proteins and lipids (191), and to the release of Ca^2+^ from the ER into the cytosol (192–194). Therefore, ER stress and Ca^2+^ efflux caused by ROS may lead to protein PTM and the formation for neo-antigens in β cells.

### Dysglycemia

As discussed earlier, increased glucose sensing by β cells during times of dysglycemia increases insulin production and secretion (75). Normal insulin secretion raises β cell ER stress (32–42), but when blood glucose rises too high, or the hyperglycemia is too prolonged, so called “glucotoxicity” further enhances β cell ER stress. At later stages of T1D, ER stress induced by glucotoxicity is thought to be a major contributor to β cell death through the terminal UPR. However, fluctuation in blood glucose levels as β cell mass is gradually lost may also induce the adaptive UPR. In this way, glucotoxicity may, long before β cell death, contribute to Ca^2+^- and PTM-dependent neo-antigen formation and therefore to autoimmune targeting of β cells.

### Inflammation

As autoreactive immune cells infiltrate the islets to target their antigens, these activated immune cells secrete pro-inflammatory cytokines. In addition, β cells themselves release additional pro-inflammatory cytokines during viral infection (195), and cellular stress (196). These inflammatory mediators initiate signaling cascades in the β cells. For example, pro-inflammatory cytokines activate NF-kB in β cells, which inhibits the expression of other transcription factors necessary for normal β cell function (197). Also, inflammatory cytokines activate c-jun N-terminal mitogen-activated protein kinase signaling, which is associated with ER stress and Ca^2+^ release (198, 199). Finally, inflammatory cytokines reduce SERCA expression, effectively preventing the return of Ca^2+^ from the cytosol to the ER and further exacerbating ER stress (197, 200). Therefore, pancreatic inflammation may lead to β cell neo-antigen formation and exacerbate autoimmune targeting of β cells.

Therefore, we hypothesize that the ER stress generated by these environmental factors may converge with the stress caused by normal physiology to allow cytosolic Ca^2+^ to cross the necessary threshold to activate PTM enzymes and generate neo-antigens long before the terminal UPR initiates apoptosis pathways. In this way, ER stress-mediated neo-antigen formation may be a common mechanism by which these environmental factors augment autoimmune targeting of β cells and hasten T1D onset.

## Conclusion

Type 1 diabetes is caused by the autoimmune targeting and destruction of pancreatic β cells. The autoreactive immune cells target many β cell proteins (Table 1) when central and peripheral tolerance fail. The mechanisms by which tolerance fails are still being elucidated, but a growing body of literature demonstrates that β cell peptides modified by Ca^2+^-dependent PTM elicit stronger responses from autoreactive T cells than their native counterparts (16, 19, 23, 28–31, 143, 145). However, the mechanisms by which these β cell peptides become modified during β cell physiology is only beginning to be explored (32, 60).

**Figure 7:**
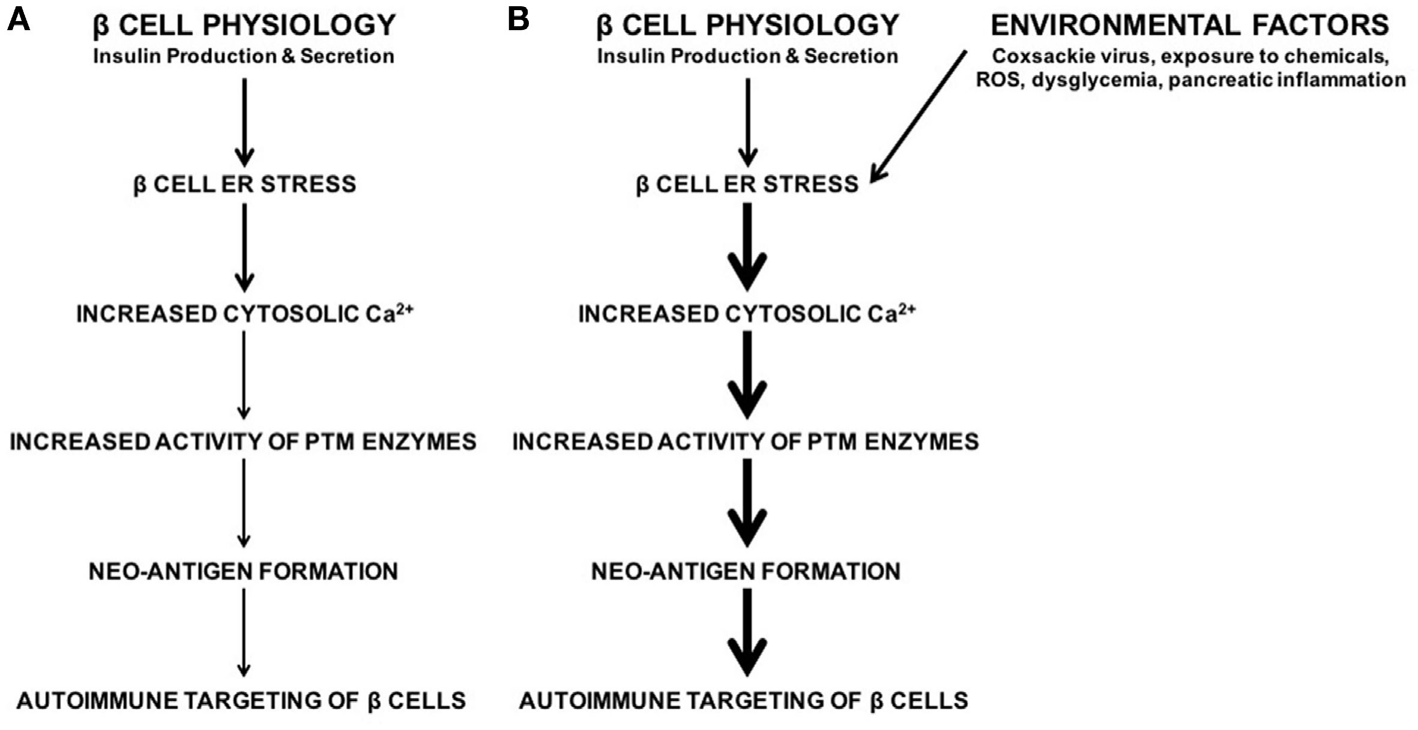
Model. **(A)** Normal β cell secretory physiology causes inherent endoplasmic reticulum (ER) stress, which in turn results in a release of Ca^2+^ from the ER into the cytosol. This ER stress and Ca^2+^ efflux lead to increased activity of Ca^2+^-dependent posttranslational modification (PTM) enzymes, formation of neo-antigens, and β cell immunogenicity (32). However, neo-antigen formation and immunogenicity due to inherent physiological ER stress may not be enough to cause type 1 diabetes (T1D). **(B)** The environmental factors associated with T1D onset cause β cell ER stress and Ca^2+^ efflux. The ER stress induced by these environmental factors cooperates with the physiological ER stress to raise cytosolic Ca^2+^ concentrations above a threshold to activate PTM enzymes, generate neo-antigens, cause autoimmune targeting of β cells, and precipitate T1D onset.

We have previously demonstrated that β cell ER stress leads to PTM-dependent immunogenicity (32). Although this ER stress may be derived from the natural secretory physiology of the β cell (32–42), inherent, physiological ER stress alone may not sufficient to precipitate T1D onset even in those individuals harboring a genetic predisposition to autoimmunity (155–157). We therefore propose a model in which β cell ER stress leads to neo-antigen formation and immunogenicity of β cells when this ER stress reaches a critical threshold. The ER stress induced by the environmental factors associated with T1D may combine with physiological ER stress to raise cytosolic Ca^2+^ above this putative threshold, allowing for the activation of PTM enzymes and the generation of PTM-dependent neo-antigens (Figure 7). This convergence of with physiological stress may explain how environmental factors hasten T1D onset.

It is important to note that, although physiological and environmental factor-derived ER stress likely occurs in the β cells of all individuals, autoimmunity predominantly occurs in the context of genetic predisposition to autoimmunity. For patients who express the MHC molecules that predispose them to autoimmunity, β cell neo-antigens generated during ER stress are presented by these MHC molecules and activate the T cells that escaped negative selection during development. The activation of these T cells ultimately leads to the autoimmune destruction of the β cells and to T1D onset. However, in those without this MHC predisposition, β cell ER stress may still result in the modification of β cell proteins without leading to disease. In these patients, these neo-antigens may not be presented by APC or may not be recognized if autoreactive T cells are correctly deleted from the repertoire during negative selection in the thymus. Therefore, β cell ER stress and the subsequent neo-antigen formation likely still require genetic predisposition to autoimmunity to lead to T1D.

Our model proposes a “threshold hypothesis” according to which cytosolic Ca^2+^ must cross a particular threshold to allow for the generation of PTM-dependent β cell neo-antigens. Additional studies are necessary to confirm the cooperation between physiological ER stress and that derived from exposure to environmental factors to reach this threshold. These studies will further advance our understanding both of how neo-antigens are formed in the β cell and the mechanisms by which environmental factors hasten disease onset. Such studies may reveal novel opportunities for therapeutic intervention to prevent or delay T1D onset in at-risk patients.

## Materials and Methods

### Mice

Mice were bred and housed under specific pathogen-free conditions at Rangos Research Center of Children’s Hospital of Pittsburgh of University of Pittsburgh Medical Center. All experiments were approved by Institutional Animal Care and Use Committee of the University of Pittsburgh.

### Cell Culture

The NIT-1 insulinoma cell line was a gift from Clayton Mathews (University of Florida) and were maintained at 37°C in a 5% CO_2_ humid air incubator, in DMEM (Invitrogen) supplemented with 10% heat-inactivated fetal bovine serum (Mediatech), 10 mM HEPES buffer (Gibco), 4 mM l-glutamine (Gibco), 200 µM nonessential amino acids (Gibco), 1 mM sodium pyruvate (Gibco), 61.5 µM β-mercaptoethanol (Sigma-Aldrich), and 100 µg/ml gentamicin (Gibco).

CD4^+^, MHC class II-restricted BDC2.5, BDC5.2.9, PD12.4.4, and BDC6.9 T cells were a gift from Kathryn Haskins (University of Colorado). T cell clones were maintained in supplemented DMEM as described previously (32, 201–203).

OT-II splenocytes were harvested and prepared in supplemented DMEM as described previously (204–208).

### Induction of ER Stress

NIT-1 cells were cultured in 25 cm^2^ tissue culture flasks (Greiner Bio-One) with various concentrations of thapsigargin or control for 1 h at 37°C or with various concentrations of tunicamycin or control for 4 h at 37°C. Before downstream analysis, the cells were washed extensively (50,000× original volume) to remove residual thapsigargin or tunicamycin, and removed from the flask with 0.05% trypsin–EDTA (Gibco).

### T Cell Assays

T cells (2 × 10^4^), NOD.*scid* splenocytes as APC (4 × 10^5^), and antigen (1 × 10^3^ dispersed NIT-1 cells) were combined in 200 µl supplemented DMEM in triplicate in 96-well flat-bottom tissue culture plates (Greiner Bio-One) and incubated at 37°C for 72 h. T_H_1 effector function was determined by measuring IFNγ secretion by enzyme-linked immunosorbent assay (ELISA).

### Splenocyte Assay

OT-II splenocytes (1 × 10^6^) were combined with antigen (1 × 10^3^ dispersed NIT-1 cells) in 200 µl supplemented DMEM in triplicate in 96-well flat-bottom tissue culture plates (Greiner Bio-One) and incubated at 37°C for 72 h as described previously (204–208). T_H_1 effector function was determined by measuring IFNγ secretion by ELISA.

### Enzyme-Linked Immunosorbent Assay

Interferon gamma from T cell assays was measured with murine IFNγ ELISA antibody pairs (BD Biosciences) as described previously (32, 202–204, 208). Absorbance was measured at 450 nm with a SpectraMax M2 microplate reader (Molecular Devices). Data were analyzed with SoftMax Pro (Molecular Devices).

### Preparation of Cell Lysates

Cells were lysed by sonication in 50 mM Tris pH 8.0, 137 mM NaCl, 10% glycerol, 1% NP-40, 1 mM NaF, 10 µg/ml leupeptin, 10 µg/ml aprotinin, 2 mM Na_3_VO_4_, and 1 mM PMSF. Protein concentration was determined by bicinchoninic acid protein assay (Thermo Fisher Scientific).

### Western Blotting

Lysates were separated by SDS-PAGE with 10% polyacrylamide gels and transferred to PVDF membranes. Membranes were blocked in 5% BSA in TBST for 1 h, and probed with antibodies to phosphorylated PERK (Cell Signaling Technology; 1:200), phosphorylated eIF2α (Cell Signaling Technology; 1:1,000), total PERK (Cell Signaling Technology; 1:1,000), and total eIF2α (Cell Signaling Technology; 1:1,000) overnight at 4°C. Membranes were washed and incubated with HRP-conjugated goat anti-rabbit (Cell Signaling Technology; 1:2,000) for 1 h. Chemiluminescence was detected with Luminata Crescendo Western HRP Substrate (Millipore) and analyzed with Fujifilm LAS-4000 imager and Multi Gage Software (Fujifilm Life Science).

### Statistical Analysis

For ELISA, data are mean IFNγ secretion ± SD or SEM (as indicated). For Western blotting, data are representative of two experiments. Densitometry data are phosphorylation levels normalized by total and relative to that in control-treated cells. Statistical significance was determined by Student’s *t*-test, and statistically significant differences are shown for *p < 0.05.

## Ethics Statement

This study was carried out in accordance with the recommendations of the Guide for the Care and Use of Laboratory Animals. The protocol was approved by the Institutional Animal Care and Use Committee of the University of Pittsburgh.

## Author Contributions

MM and JP contributed to the composition of this manuscript.

## Conflict of Interest Statement

The authors declare that the research was conducted in the absence of any commercial or financial relationships that could be construed as a potential conflict of interest.

## Abbreviations

Aire, autoimmune regulator; APC, antigen-presenting cell; ATF6, activating transcription factor 6; ATP, adenosine triphosphate; Ca^2+^, calcium; CHgA, chromogranin A; DRiP, defective ribosomal product; EAE, experimental autoimmune encephalomyelitis; ER, endoplasmic reticulum; GAD65, glutamic acid decarboxylase 65; GFP, green fluorescent protein; GRP78, 78 kDa glucose-regulated protein; IA-2, tyrosine phosphatase-like insulinoma antigen 2; IAPP, islet amyloid polypeptide; ICA69, islet cell autoantigen 69; IGF-2, insulin-like growth factor 2; IGRP, islet-specific glucose-6-phosphatase catalytic subunit-related protein; IFNγ, interferon gamma; IP_3_R, inositol 1,4,5-trisphosphate receptor; IRE1, inositol-requiring protein 1; JNK, c-jun N-terminal kinase; MAP, mitogen-activated protein kinase; MHC, major histocompatibility complex; mTEC, medullary thymic epithelial cell; NET, neutrophil extracellular traps; NF-κB, nuclear factor kappa-light-chain-enhancer of activated B cells; NOD, non-obese diabetic mouse; PAD2, peptidylarginine deiminase 2; PDI, protein disulfide isomerases; PERK, protein kinase RNA (PKR)-like ER kinase; Phogrin, phosphatase homolog of granules from rat insulinomas; PTM, posttranslational modification; ROS, reactive oxygen species; RyR, ryanodine receptor; SERCA, sarco/endoplasmic reticulum Ca^2+^ ATPases; SNP, single-nucleotide polymorphisms; T1D, type 1 diabetes; Tgase2, tissue transglutaminase 2; UPR, unfolded protein response; ZnT8, zinc transporter 8.
